# Vancomycin Capped with Silver Nanoparticles as an Antibacterial Agent against Multi-Drug Resistance Bacteria

**DOI:** 10.15171/apb.2017.058

**Published:** 2017-09-25

**Authors:** Mahsa Esmaeillou, Gholamreza Zarrini, Mohammad Ahangarzadeh Rezaee, Javid Shahbazi mojarrad, Ali Bahadori

**Affiliations:** ^1^Department of Biology, Faculty of Natural Sciences, University of Tabriz, Tabriz, Iran.; ^2^Immunology Research Center, Tabriz University of Medical Sciences, Tabriz, Iran.; ^3^Department of Medicinal Chemistry, Faculty of Pharmacy, Tabriz University of Medical Sciences, Tabriz, Iran.; ^4^Department of Medical Microbiology, Sarab Faculty of Medical Sciences, Sarab, Iran.

**Keywords:** Vancomycin, Silver nanoparticles, Antibacterial agent, Multi-drug resistance bacteria

## Abstract

***Purpose:*** Many antimicrobial medications are available to combat infections. However, the indiscriminate use of antibiotics has produced antibiotic resistance in the case of many bacterial pathogens. This study focuses on the development of nanoparticles (NPs) that enhance the in vitro antibiotic activity of vancomycin against multi-drug resistant (MDR) organisms.

***Methods:*** Spherical shaped thioglycolic acid-stabilized silver nanoparticles (TGA-AgNPs) were prepared by using a simple chemical reduction method. Then, vancomycin was conjugated to the terminal carboxyl of TGA in the presence of N-Hydroxysuccinimide (NHS) and N-(3-dimethylaminopropyl)-N’-ethylcarbodiimide hydrochloride (EDC). Afterwards, the antibacterial activity of these nanoconjugates was examined by using the minimum inhibitory concentration (MIC) assay against MDR bacteria.

***Results:*** The rate of vancomycin bound to the AgNPs was 19.6%. The MIC values of vancomycin (Van)-capped AgNPs against tested pathogens were in the range of (3.2, 1.6, 0.8, 0.4, 0.2, 0.1, 0.05, and 0.025 µl/ml). The MIC was 0.1 µg/ml for VRE, MIC≤0.02 µg/ml for MRSE, and 0.05 µg/ml for S. aureus. The MIC corresponded to the MBC for all bacterial species.

***Conclusion:*** This study indicated that some antimicrobial agents like vancomycin can be conjugated with AgNPs. This can lead to increased antimicrobial activity against MDR microorganisms.

## Introduction

The emergence of multi-drug resistance (MDR) among various bacterial pathogens has become a serious problem for the medical world, limiting the choice of antibiotics. Any use of antibiotics can increase selective pressure on bacteria. This allows resistant bacteria to survive and susceptible bacteria to die.^1^ As resistance to antibiotics becomes more common, a greater need for alternative treatments is on the rise. Advances in nanotechnology have opened up new horizons in nanomedicine, allowing the synthesis of nanoparticles (NPs) that can be assembled into complex architectures such as antibiotic structures.^2^

Today, vancomycin resistance in gram-positive cocci is a growing global problem. In the early 1950s, vancomycin was used as the primary antibacterial agent for the treatment of infections caused by methicillin-resistant *Staphylococcus aureus* (MRSA). Development studies on vancomycin in 1956 revealed that vancomycin resistance in staphylococci was difficult to induce *in vitro.*^3^ The first Vancomycin-resistant Enterococci (VRE) strains appeared in 1988. Since then, more and more glycopeptide-resistant strains have been found showing that these bacteria may potentially transfer the genes that make them resistant to vancomycin to other species of bacteria.^4^ Progress in the development of new drugs to tackle VRE has been improved by using nanomedicines.

Silver nanoparticles (AgNPs) have found plenty of applications as antibacterial agents because of their biological activities and safety. Their synthesis is generally carried out through the reduction of Ag3+ using inorganic agents or through biogenic approaches. Numerous functional bioconjugate NPs such as the single-strand DNA,^5^ anti-bodies, ciprofloxacin,^6^ phospholipids, and various proteins capped NPs, have emerged in the past decade for the development of surface modification techniques on chemical structure, allowing useful application in the fields of drug design, drug delivery, biosensors, and so on.^7^ In our investigation, vancomycin was conjugated to the terminal carboxyl of Thioglycolic acid (TGA). TGA was used as a bridge linker and then its antimicrobial activities against VRE and other MDR pathogens were determined.

## Materials and Methods

### Chemicals

AgNO_3_ (93.8%), TGA (98%) and N-Hydroxysuccinimide (NHS) were purchased from Merck, Germany. 1-(3-dimethylaminopropyl)-3-ethylcarbodiimide hydrochloride (EDC) and 3-(N-Morpholino) propanesulfonic acid (MOPS) buffer were purchased from Sigma, USA. Sodium borohydride (NaBH_4_) was purchased from DAE Jung chemicals and vancomycin was purchased from Dana Pharmacy Company, Iran.

Doubly distilled and deionized water was used as the solvent for preparing the stock solutions of all reagents. Buffer and all other chemicals were prepared according to standard laboratory procedures and the manufacturer’s guidelines.

### Silver nanoparticle preparation 

Spherical TGA-stabilized AgNPs were synthesized by the reduction of AgNO_3_ with NaBH_4_. For this purpose, in an Erlenmeyer flask, 2.5 mL of 10^-2^ mol.L^-1^ AgNo_3_ was added to75 mL deionized water. Afterwards, 5mL of 10^-2^ mol.L^-1^ TGA was appended to the solution while stirring vigorously. After the colour of the solution changed yellow or greenish yellow, 2.5 mL of 10^-2^ mol.L^-1^ Potassium iodide (KI) was dropped into the solution slowly, yielding a yellow AgI colloid. In the next step, 20 mg of NaBH_4_ was added to this solution, and mixed for 20 minutes. Finally, the colour of colloidal solution changed to brown and AgNPs were obtained. To remove the residue stabilizer and other ions, the obtained AgNPs were centrifuged at 12000 rpm for 20 minutes and washed thrice with doubly distilled water. The obtained NPs were re-dispersed in deionized water by sonication and preserved for other experiments.

### Scanning electron microscopy

SEM samples were prepared by adding 2 mL of NP suspension on the glass surface and allowing it to air-dry. They were imaged with MIRA3 TESCAN SEM at an operating voltage of 10.0 kV.

### Conjugates preparation

Conjugates were prepared dispersing Ag-NPs in 25 mL MOPS buffer (50mM, pH 6.5) in the presence of NHS (0.34 mg) and EDC (0.34 mg). After 30 minutes, 20 mg of vancomycin was added to mixture and stirred at room temperature for 24 hours under vigorous mixing. Excess vancomycin and other chemicals were eliminated by removing the supernatant after centrifugation at 12000 rpm for 20 minutes, and resuspended in deionized water and stored at 4 °C. To prepare a stock solution of vancomycin 0.2mg of this drug was dissolved in 100 mL deionized water.

### Bacterial strains and Antimicrobial testing conditions

Bacterial strains and their relevant attributes are mentioned in Table 1. Strains were isolated from children hospitalized in the Tabriz Pediatric Hospital and maintained in Brain-Heart infusion (BHI) medium. To determine the Minimum Inhibitory Concentration (MIC) of nanoparticles and (Van)-capped Ag nanoparticles, strains were grown in Mueller Hinton Broth with aeration at 37°C for 24 hours.

**Table 1:** Bacterial strains used in our study.

**Strain**	**Relevant phenotype**	**source**
*Staphylococcus aureus*	Methicillin Resistant, Gram positive, Vancomycin intermediate	Surgical wound
*Enterococcus faecalis*	Vancomycin resistant, Gram positive, MDR	Urine sample
*Staphylococcus epidermidis*	Vancomycin intermediate, Gram positive	Blood cultures
*Pseudomonas aeruginosa*	MDR, Gram negative	Blood cultures
*Escherichia coli*	MDR, Gram negative	Urine sample

The effects of nanoparticles, pure vancomycin and (Van)-capped AgNPs on individual bacterial isolates were determined according to the following protocol. First, the plates were sterilized. Then, 200 µl of Mueller Hinton Broth was added to each well. At the first well of the 96-well plate (Van)-capped AgNPs with a concentration of 3.2 µg/ml were dispersed in sterile Mueller Hinton Broth with a final volume of 400 µl. At the next step, 200 µl of the first well solution was poured into the second well resulting in half concentration of Ag conjugates. This process was iterated as far as the penultimate well. Thus, different antibiotic concentrations (3.2, 1.6, 0.8, 0.4, 0.2, 0.1, 0.05, and 0.025 µl/ml) were obtained from the base antibiotic stock solution. The bacterial suspensions for each bacterial isolate were prepared and adjusted to 0.5 McFarland (1.5 ×10^8^ CFU/ml) standard. Then, 10 µl of each suspension was added to the wells to obtain a final concentration. The last well was filled with only Mueller Hinton Broth medium and used as a control well. Then all the wells were kept at 37°C for 24 hours and the growth of bacteria was checked. The MIC of the nanoconjugates was of the lowest concentration and showed no growth. Similarly, this protocol was performed for pure vancomycin and AgNPs. The results were then compared.

In this study, for the purpose of calculating the amount of loading, the calibration curve was plotted and the obtained linear equation was solved.

## Results and Discussion

### Formation of the silver nanoparticles

**Figure 1:**
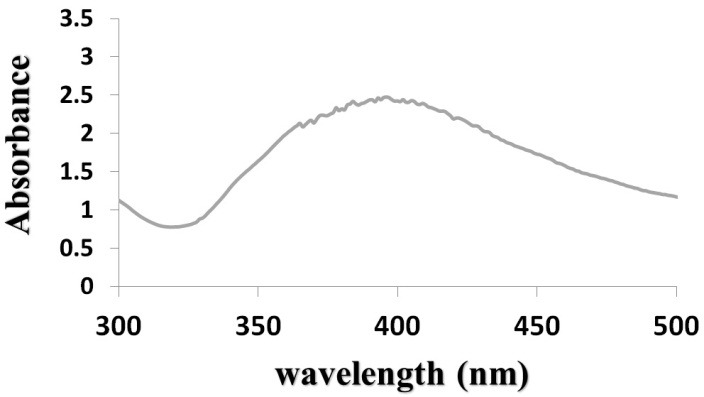
The UV–visible spectroscopy of silver nanoparticles showed the Plasmon resonance peak noted around 398 nm.

**Figure 2:**
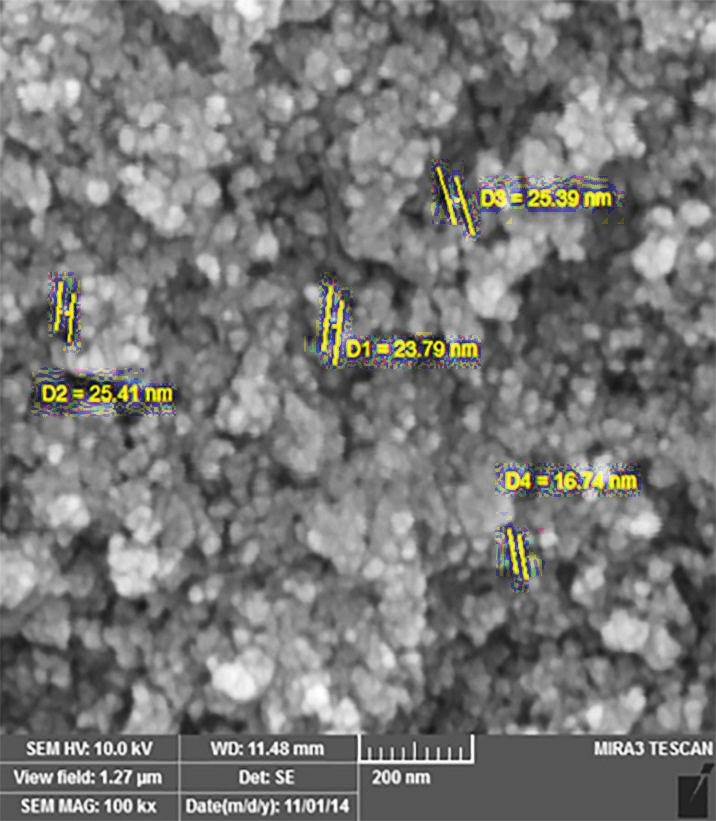
The scanning electron micrograph of silver nanoparticles showed the average size of silver nanoparticles was about 16-25 nm.

**Figure 3:**
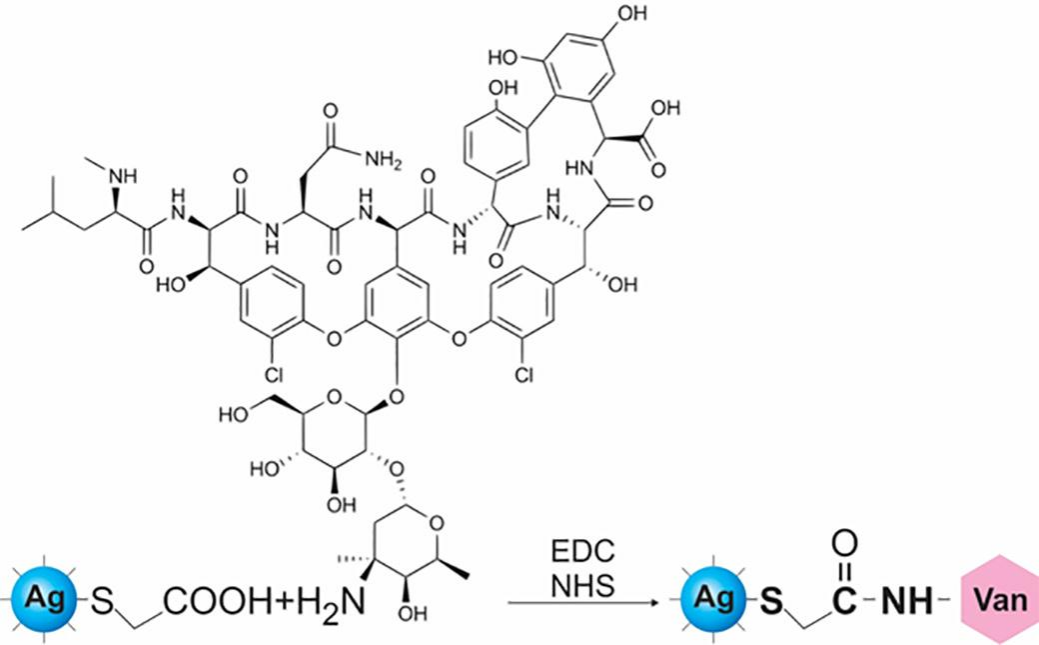
Reaction scheme for thioglycolic acid stablazed Ag silver nanoparticles capped with vancomycin by amid bounds.

In the first phase of experiments, AgNPs were prepared by wet chemical reduction method. The formation of TGA-stabilized AgNPs was traced by the UV-visible spectra and scanning electron microscopy (SEM). Figure 1 shows the UV-visible absorption of AgNPs. A very significant absorption band at about 398 nm was observed. This absorption could indicate the formation of AgNPs. The spectroscopy method was used to prove the changes of surface plasmon resonance. AgNPs were thus produced. Figure 2 shows the SEM micrograph of AgNPs. Conjugation of vancomycin to the NPs via condensation reaction in the presence of EDC and NHS to activate the carboxyl group is shown in Figure 3. In this chemical reaction, TGA worked as a ‘bridge’ molecule for binding the AgNPs with vancomycin by amid bond. The surface carboxylic acid groups of TGA activated with EDC and NHS to form the NHS ester and substitution of the NHS ester with an amino group on the gentamicin to form an amide bond.^6^

**Figure 4:**
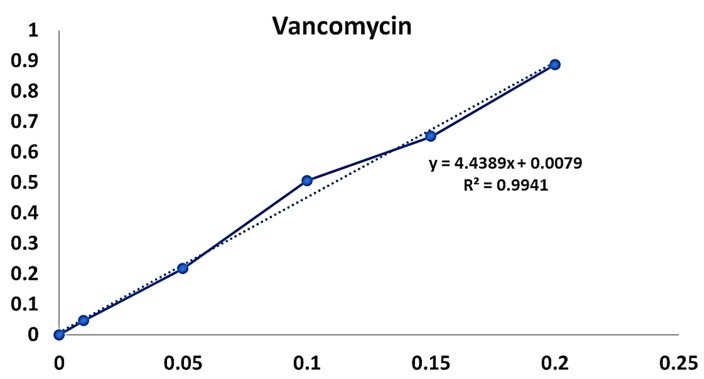
Calibration curve of serial dilutions of vancomycin.

The amount of vancomycin bound to the AgNPs was 19.6% and calculated using a calibration curve (Figure 4).

### In vitro antimicrobial activity 

Bacteriological examinations were performed in Mueller Hinton Broth liquid systems. The conjugates exhibited antimicrobial activity against MDR bacterial strains. The MIC of pure vancomycin and AgNPs and (Van)-capped AgNPs for these bacteria was calculated as the lowest concentration at which bacteria growth was inhibited. The MIC of pure vancomycin for gram-negative strains was ≥3.2µg/ml. When pure AgNPs were employed, the MIC was 0.8 µg NP per ml for VRE, 0.4 µg NP per ml for MRSE, and 0.8 µg per ml for *S. aureus*. Furthermore, when NPs are employed as a capping agent in combination with vancomycin, MIC decreased considerably. In this condition, the MIC was 0.1 µg/ml for VRE, MIC≤0.02 µg/ml for MRSE, and 0.05 µg/ml for *S. aureus*. The MIC corresponded to the MBC in all bacterial species. The results are shown in Table 2.

**Table 2:** The effects of nanoparticles on Gram-positive and Gram-negative Multidrug-resistant bacteria

**Strain**	**AgNP (μg/ml)**	**Vancomycin (μg/ml)**	**Van-AgNP (μg/ml)**
*Staphylococcus aureus*	0.8	MIC≥3.2	0.05
*Enterococcus faecalis* (VRE)	0.8	MIC ≥3.2	0.1
*Staphylococcus epidermidis* (MRSE)	0.4	MIC ≥3.2	MIC≤0.02
*Pseudomonas aeruginosa*	1.6	MIC ≥3.2	MIC ≥3.2
*Escherichia coli*	1.6	MIC ≥3.2	MIC ≥3.2

Direct comparisons between our findings in this research and those from other works are complicated. Insomuch, the physiochemical characteristics of the AgNPs utilized in several studies were different. On the other hand, the protocols used to recognize bactericidal activities are different from other investigations. We used a chemical reduction method to synthesis our AgNPs. In the next step for the measurement of surface plasmon resonance and to prove the formation of AgNPs, we used UV-visible spectroscopy. In our study, we obtained the UV-visible absorption of the AgNPs around 398 nm that shows the plasmon resonance peak. Smaller NPs primarily absorb light and have peaks near 400 nm, while larger spheres exhibit increased scattering and have peaks that broaden and shift towards longer wavelengths (known as red-shifting).^8^ The shape and size of AgNPs and the context in which the assay is performed are important factors in its capacity to annihilate superbugs. In two previous studies, researchers used antibiotics and AgNPs together and found a synergy resulting in better antibacterial efficacy. However, in these cases, there was no effort to graft antibiotics to AGNPs.^9,10^ Albeit in another investigation, one author suggests that, the vancomycin in conjunction with AgNPs can be an effectual inhibitor for gram-negative bacteria such as *E. coli.*^11^ However, in our investigation, the result was different, our findings showed that, vancomycin can be conjugated with AGNPs. It can also enhance antibacterial activates against gram-positive bacteria, but not gram-negative bacteria, which can be justified because it is associated with the outer membrane of gram-negative bacteria. GU et al. worked on the MIC of vancomycin-bound gold NPs and they showed that this combination has an inhibitory effect on the growth of *Enterococcus* spp.^12^ It was observed that the vancomycin-bound AgNPs had increased bactericidal activity compared to pure vancomycin. NP synthesis could be done with different ligand stabilizers. TGA has strong capping eligibility on the surface of AgNPs and is an applicable stabilizer. In this research, TGA was used as a stabilizer for AgNPs. This produced a carboxylic agent which helped to create chemical bonds with gentamicin. To do this, an amide bond was chosen due to its chemical stability. The results showed that *in vitro* antibacterial activity against gram-positive bacteria by binding AgNPs onto the surface of vancomycin, kill MDR bacteria.

## Conclusion

This investigation demonstrated that vancomycin can be conjugated with AgNPs to enhance antibacterial activity against gram-positive bacteria, but not gram-negative bacteria. *In vitro* bactericidal examinations showed that the (Van)-capped with AgNPs exhibited substantial activity against VRE and MRSE. According to these findings, this nanoconjugate system may provide a frugal method for the development of a new generation of effective antibacterial agents.

## Acknowledgments

This study was supported by the University of Tabriz, Iran. This is a MSc thesis by a student registered at the University of Tabriz. The authors are very grateful to the Department of Medicinal Chemistry, Faculty of Pharmacy, Tabriz University of Medical Sciences laboratory and Pashmineh Biochemistry Research Centre.

## Ethical Issues

Not applicable.

## Conflict of Interest

The authors have no conflict of interest that is directly relevant to the content of this manuscript.
